# National Evaluation of Canadian Multi-Service FASD Prevention Programs: Interim Findings from the Co-Creating Evidence Study

**DOI:** 10.3390/ijerph16101767

**Published:** 2019-05-18

**Authors:** Deborah Rutman, Carol Hubberstey

**Affiliations:** 1 Principal, Nota Bene Consulting Group, Victoria, BC V8R1P8, Canada; carolmarie@shaw.ca; 2 School of Social Work, University of Victoria, Victoria, BC V8W2Y2, Canada

**Keywords:** FASD, FASD prevention, program evaluation, multi-service delivery, integrated programs, multi-site evaluation

## Abstract

Since the 1990s, a number of multi-service prevention programs working with women who have substance use, mental health, or trauma and/or related social determinants of health issues have emerged in Canada. These programs use harm reduction approaches and provide outreach and “one-stop” health and social services on-site or through a network of services. While some of these programs have been evaluated, others have not, or their evaluations have not been published. This article presents interim qualitative findings of the Co-Creating Evidence project, a multi-year (2017–2020) national evaluation of holistic programs serving women at high risk of having an infant with prenatal alcohol exposure. The evaluation utilizes a mixed-methods design involving semi-structured interviews, questionnaires, focus groups, and client intake/outcome “snapshot” data. Findings demonstrated that the programs are reaching vulnerable pregnant/parenting women who face a host of complex circumstances including substance use, violence, child welfare involvement, and inadequate housing; moreover, it is typically the intersection of these issues that prompts women to engage with programs. Aligning with these results, key themes in what clients liked best about their program were: staff and their non-judgmental approach; peer support and sense of community; and having multiple services in one location, including help with mandated child protection.

## 1. Introduction

For women, problematic substance use is often interconnected to a host of issues including current and/or historic experiences of violence, intimate partner violence, trauma, inadequate or unsafe housing, homelessness, poverty, child welfare involvement, maternal–child separation, and mental health and physical health problems [1,2,3,4]. In addition, women who are pregnant and have substance use issues along with other complex circumstances commonly face a number of personal, programmatic and systemic barriers to accessing services. These include: stereotyping and judgements; lack of mental health support; and avoidance of services due to fears of child welfare authorities [5]. Poor access to community services as a result of lack of transportation and/or child care represent additional barriers [3,6,7]. In order to reduce the likelihood of an alcohol-exposed pregnancy, helping women overcome pervasive barriers becomes critical [8]. Accordingly, prevention responses in relation to high risk and vulnerable women need to align with what is known about the range of issues and challenges associated with prevention of fetal alcohol spectrum disorder (FASD).

For some women who use substances, pregnancy is pivotal, a time when they are willing to contemplate making life changes. Indeed, the research literature suggests that women who are pregnant will respond to supports that are aimed at improving their health, including efforts to decrease or stop substance use or to increase their safer use of substances [9,10,11]. In addition, there is strong evidence that outcomes for mothers and infants improve when accessible, women-centered substance use services or treatment are offered in conjunction with prenatal care [8,12,13,14].

### 1.1. Emergence and Evaluation of FASD Prevention Programs

A number of community-based prevention programs emerged in Canada in the early 1990s to address the health needs of pregnant and early parenting women with substance use issues and other complex challenges. Sheway in Vancouver and Breaking the Cycle in Toronto led the way; similar programs followed, all of them unique yet designed to address women’s holistic needs through outreach and single access drop-in services and by employing relational, trauma-informed approaches [4].

It is worth noting that many women who use substances during pregnancy are polysubstance users [15]. Moreover, research has shown that they tend to be selective about which substances to cease or reduce using and which to continue to use [15]. Consequently, the community-based prevention programs that have been leaders in the field in Canada focus on problematic substance use more broadly, within the context of a social determinants of health context and women’s lived experiences [12]. 

Establishing the effectiveness of such programs has been challenging, in part because they are community-based and/or lack resources, time, knowledge, or expertise to carry out an evaluation of their programs, or they are small programs that have not been the focus of larger studies [16]. As such, many FASD prevention services have not been formally evaluated, have not had the resources or opportunities to conduct a multi-year evaluation, or their evaluations have not been published [17]. 

That said, there are a few notable exceptions, namely Sheway [18,19], Breaking the Cycle [4,5,20,21], the Healthy, Empowered and Resilient (H.E.R.) program [22,23,24], and HerWay Home [25]. Findings from these evaluations demonstrate that these programs can help women:

- Experience better and earlier access to prenatal care
- Reduce their substance use
- Improve their health and wellness outcomes and those of their children
- Retain and/or regain custody of their children
- Develop new social/support networks and friendships
- Acquire parenting skills

Moreover, the research and evaluation literature indicates that services that employ non-judgmental, relationship-based, trauma-informed and harm reduction approaches and that also understand and seek to remove social environmental barriers to participation, such as transportation, child care, meals, stigma, and fear of child removal, have been found to be most effective in reaching vulnerable pregnant and parenting women with substance use issues [4,5,8,26]. These approaches recognize and accept the pace and type of change women are able to make and the strategies women use to cope with difficult life circumstances [5,27]. 

While these approaches have evidence, additional study is needed to understand their implementation in community settings, the relative importance of various program components especially in light of different community and cultural contexts, and the linkage to other levels of health care and social supports for women and children. Indeed, these were the conclusions of a recent national gap analysis related to FASD prevention, which found that evaluation of specialized, holistic FASD prevention programs was a key gap in research, and moreover, that a multi-site evaluation of FASD prevention programs would be a valuable next step [28]. To address this oversight in the research literature, a multi-site evaluation of “wrap-around” FASD prevention programs serving women at risk was conceived.

### 1.2. Co-Creating Evidence Evaluation Study

The Co-Creating Evidence: National Evaluation of Multi-Service Programs Reaching Women at Risk (CCE) project is a multi-year national evaluation of eight different holistic programs serving women at high risk of having an infant with prenatal alcohol exposure. The study is the first of its kind in Canada. The evaluation, which runs from 2017 to 2020 and is funded by the Public Health Agency of Canda, aims to: bring together many of Canada’s community-based multi-service FASD prevention programs to share promising approaches and practices; undertake a prospective, multi-site evaluation on the effectiveness of FASD prevention programming serving women with substance use and complex issues; and identify characteristics that make these programs successful. The project’s primary research questions are:

- What are common elements of these diverse, multi-service programs?
- How do the programs reflect their community’s context?
- What program components are most helpful from women’s perspectives?
- What are the programs’ impacts and outcomes for clients and their children?

The study is employing a mixed-methods design involving: two rounds of semi-structured interviews and questionnaires (taking place in April–July 2018 and September–December 2019) with clients, focus groups and interviews with programs’ staff and service partners; output/program data; and client intake/outcome “snapshot” data. Both quantitative and qualitative data are being gathered in order to obtain an in-depth description of each of the participating program’s theoretical foundation, administration, activities and services, strengths, challenges, and outcomes from the perspective of clients, staff and service partners. 

**Figure 1:**
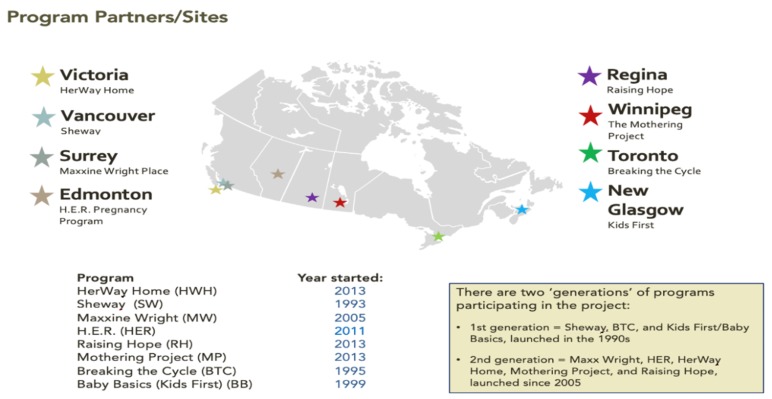
The Co-Creating Evidence project program partners.

Figure 1 shows the programs taking part in the study and their location; Box 1 provides capsule desciptions of the programs. As there are few community-based, multi-service prevention programs serving pregnant and parenting women who use substances and have other complex, social determinants of health issues in Canada, the researchers invited all such programs known to them to participate in the study as program partners, and all programs accepted. The researchers also sought and were largely successful in having regional representation across Canada, though at the time that the study was being developed, there were no community-based multi-service FASD prevention programs in Quebec or in Canada’s three northern territories.

Seven of the eight programs specifically target at-risk pregnant or early parenting women struggling with substance use or who are early in their recovery. One program focuses on serving women who are at risk by virtue of being young and possibly social isolated; while substance use may be an issue, it is not the primary focus, although given the very limited range of services available in the region, it is an issue that comes up with increasing regularity. While these programs are doing FASD prevention work, because they approach the women’s issues holistically and employ a social determinants of health lens, and because of the stigma associated with FASD and alcohol use during pregnancy, the programs typically do not depict themselves as FASD prevention programs. 

This article shares preliminary findings from the study, based on the first round of data gathered between April and December 2018. To begin, the article presents the project’s Theory of Change, which was collaboratively created by the program sites and the project team as part of the developmental phase of the study. In program evaluation, the Theory of Change is a useful tool for depicting how a desired social change occurs as a result of the service/program/intervention, by showing the interconnections between a program’s philosophy/approaches; activities; and anticipated outcomes at the client/family, community, and systems levels. Then, informed by the study’s first research question, the article briefly lists the services and activities offered across the eight programs; as well, as means to increase understanding of who are the women served by these programs, the article describes women’s situations at intake, emphasizing the multiple, complex issues that women seek to address when accessing these prevention programs. Next, the article highlights how clients use the services. Lastly, guided by the study’s third research question, the article presents qualitative findings based on the client interviews regarding what women like best about their program. 

Interim qualitative findings in relation to the connection between what women were hoping to gain from their program and what they perceived as the most significant change in their life as a result are presented in a separate article. Findings based on the second round of on-site data collection will be presented in future publications, including a more in-depth analysis of the full 18 months of program output-based and client-based data compiled by program sites.

Box 1 **Capsule descriptions of Co-Creating Evidence study’s program sites.**

**HerWay Home (HWH)** offers drop-in and outreach support, on-site wellness and prenatal/post-natal groups as well as other health/medical services for women and their children, through a combination of program staff and in-kind support from health/medical partners. *Women can participate in HWH until their child is approximately three years old.*

**Sheway (SW)** offers a broad range of on-site health/medical and social services/supports through its program staff and via partnerships. Housing that is available to Sheway clients is located on the third floor of the building (operated by the YWCA through its Crabtree program). Sheway also has a health clinic. Voluntary child welfare services are provided on-site through a partnership agreement with the provincial Ministry. *The length of time that women can participate in Sheway is flexible and not set by the child’s age*.

**Maxxine Wright (MW)** offers health and social supports through co-location with Atira Women’s Resource Society, which operates transition housing and second stage housing on-site. Atira offers most of the social programming, with participation from MW. Health/medical care is provided by MW. Child welfare and income assistance services are provided on-site through a partnership agreement with relevant provincial Ministries. *Women can participate in MW until their child enters school.*

**Healthy, Empowered, and Resilient (H.E.R.)** is located within the Boyle Street Community Services, which provides an array of social, mental health, family, and cultural services in Edmonton’s downtown core. H.E.R. provides outreach to highly street-involved clients; through its staffing and partnership with Boyle McCauley Health Centre, H.E.R. clients have access to prenatal care and post-natal support. *Women can participate in H.E.R. until six months post-partum.*

**Raising Hope (RH)** is a residence-based program that is located in an 18-unit apartment building (purchased by a non-profit housing society for the program’s exclusive use). A range of health/medical, social/cultural supports and programming, including child care, is offered on-site; residents are required to take part in daily programming. *Women and their children can stay for 18 months*.

**The Mothering Project (MP)** is a program of Mt Carmel Clinic and is co-located with the Clinic. Through its staffing and partnerships, the MP offers a broad range of drop-in, outreach and on-site supports and health/medical service along with a dedicated space for cultural ceremony. A licensed day care is co-located with MP with spaces set aside for program clients. *Women can participate in the MP until their child reaches the age of five.*

**Breaking the Cycle (BTC)** is one of the first FASD prevention programs in Canada. The program provides children’s developmental assessment and mental health services with wrap-around services for women. Each woman is connected with a counsellor and each child is connected with a Child Development Worker. *Women can stay at BTC until their child is six years old.*

**Baby Basics (BB)** is a weekly drop-in parenting program operated by Kids First Family Resource Program, for women under age 25 and their children aged 0–6. Although not specifically directed at women who are using substances, there are very few such options available to women in the region. BB offers a safe place for women to access support and talk about a range of issues. *Women can participate in BB until their infant is one year old.*

## 2. Methodology and Materials

### 2.1. Study Design

As a multi-site evaluation, this mixed-methods study is being guided by collaborative and participatory principles [29], including the principles of “fostering meaningful relationships” (with program staff and stakeholders), “developing a shared understanding of the program”, “promoting appropriate participatory processes”, and “promoting evaluative thinking” [30,31]. As a means to put these principles into practice, the project team convened an introductory face-to-face meeting with the eight Program Managers/Coordinators. Since that time, bi-monthly web-based teleconferences have been held with sites to discuss key issues related to data collection and analysis, and to solicit the programs’ feedback regarding interim project findings, draft reports and knowledge translation. Web-based meetings with Program Coordinators/Managers are also an opportunity for the programs to exchange information regarding promising practices, programming, emerging challenges and contextual issues of significance (e.g., the current opioid crisis and its impact on clients, staff and program delivery). In addition, at the beginning of the project, the researchers engaged a national Advisory Committee comprised of people with expertise in policy, programming, research and evaluation related to FASD. The Advisory Committee has been meeting 2–3 times a year to provide guidance and feedback on key facets of the project including data collection, analysis and knowledge translation.

The evaluation study received ethics approval from the University of British Columbia Office of Research Ethics (H17-02168), the Fraser Health Authority, the Vancouver Costal Health Authority, and the Island Health Authority. All study participants provided informed consent to participate in the study. As well, the client and output data gathered by the program sites are fully de-identified and are being transmitted to the project team via a secure file transfer program, where the data are combined into one SPSS database for data management and analysis. Prior to the first transfer, a Data Transfer Agreement discussing the parameters governing the collection, transmission, storage, security, analysis, re-use, archiving, and destruction of data was signed by each site.

### 2.2. Data Collection Processes and Instruments

In this study, data are being gathered in two different ways: 

(1) by the program sites, wherein quantitative output data and client-based data are being compiled and sent to the project team quarterly for a total of 18 months (April 2018 to September 2019); the Client Database contains demographic and high-level outcome indicators for each woman attending the program during the quarter, including related to housing, birth outcome, substance use, and child welfare-related outcomes; and

(2) by the project team, wherein two researchers visit each program twice to conduct face-to-face semi-structured interviews, focus groups, and questionnaires, supplemented by telephone interviews when necessary. 

The first round of site visits and in-person data collection took place between April and July 2018; the second round will take place in the fall of 2019. Following that, quantitative and qualitative data analyses based on the full data set will be undertaken. 

### 2.3. On-Site Data Collection by Program Team

The research team conducted individual interviews and fixed-choice questionnaires with clients in a private and comfortable office at each program site. The qualitative interviews were conducted using a “guided conversation approach” [32], which enabled and encouraged women to speak freely about issues and experiences of significance to them. All clients participating in the study were provided an honorarium for completing the interview and questionnaire, which together took approximately 30–40 min. As well, program staff were available afterwards to address any concerns that clients had about the interview. Individual interviews with service partners were carried out in person or by telephone. A focus group was held with staff at each of the programs, along with individual interviews with the Program Coordinator or Manager at each program.

The Interview Guides were custom-created for this study. The Interview Guide for Clients contained open-ended questions focusing on: how the woman first learned about the program and what she hoped to get out of her involvement with it; her life situation at the point at which she first engaged with the program; her satisfaction with the program (e.g., what she liked most about the program, what she did not like, and what she would change); and what was most important to her about the program. The interview with clients also covered perceived impacts of the program and included a modified version of the Most Significant Change (MSC) technique [33], which is a story-telling process that has been used in evaluation of Indigenous family-serving projects. Using the MSC technique, informants are asked to share a story or example of “the most significant change that happened” as a result of the program. As well, the interview contained open-ended questions pertaining to the client’s use of the program’s various services and activities. A full discussion of the data based on both rounds of on-site interviews will be forthcoming in 2020. 

The Questionnaire for Clients, utilizing a 5-point Likert-type scale, was included in the interview process. While the questionnaire was created specifically for this study, it also included standardized questionnaire items that have been used in evaluations of trauma-informed and/or harm reduction focused programs [34]. Findings based on the Questionnaire for Clients will be included in forthcoming publications. 

Prior to launching formal data collection, the research team pilot tested the interview guides and interview process at four sites to gain feedback from clients and staff regarding the process and the questions, including whether clients were comfortable with answering potentially difficult questions about their lives. 

### 2.4. Participants and Sampling Approach 

A total of 228 people took part in the first round of in-person (on-site) data collection for the study, including:

- 125 program participants/clients (of whom 123 completed the Questionnaire for Clients);
- 61 program staff; and
- 42 service partners

For the purposes of this article, only client data are presented and discussed.

The number of interviews with clients varied across sites, from *n* = 32 at one of the large sites to *n* = 8 at two of the sites; there were three sites at which more than 20 clients were interviewed and five sites at which 8–11 women were interviewed. The variation in the number of interviews conducted per site reflected the size and scale of the programs and was roughly proportional to the number of clients per site. Events outside of the program, including crises in the community and/or in clients’ lives also impacted response to the invitation to take part in the study.

Eligibility criteria for client participation in the interview/questionnaire were as follows: (1) women had to be accessing services from the program in the month of data collection; (2) women had to be 16 years or older; and; (3) women had to be English-speaking. Clients were recruited and informed by program staff approximately one month in advance about the research team’s site visit and the opportunity to participate in a face-to-face interview. Posters announcing the interviews were also displayed in program space and announcements were made at meetings and groups. The recruitment material included information about confidentiality and anonymity. For clients, the study employed a voluntary sampling approach, as Program Coordinators and the research team believed it important for all clients who wanted to take part in an interview and who were available during the researchers’ site visit to have the opportunity to do so.

A nominated sampling approach was used to create the sample of service partners; program staff at all sites provided the researchers with contact information for each program’s closest service partners. The researchers then conducted face-to-face or phone interviews with service partners, which lasted from 30 to 90 min. Interviews and focus groups with program staff were conducted in person and were from 1 to 2 h in duration.

### 2.5. Data Analysis

For the quantitative client and output data, a frequency analysis was conducted to describe the program activities and outputs, the client’s pregnancy, substance use, child welfare and housing status at intake into each program. The analysis was conducted in IBM SPSS 26 (SPSS Inc., Chicago, IL, USA). 

As the interviews with clients, staff, and service partners involved open-ended questions, qualitative data analysis techniques were used, and qualitative data analysis software (NVivo12, QSR International Pty Ltd., Melbourne, Australia) was utilized to facilitate the analyses. In keeping with these techniques, written transcripts from all interviews were read multiple times by the researchers to begin the process of identifying themes and issues. Initially, each researcher coded the transcripts separately and identified preliminary themes inductively. The team highlighted naturally occurring patterns in the data, which formed the basis of the thematic analysis [35,36]. As means to strengthen the study’s rigor, the researchers engaged in numerous discussions wherein they presented and reviewed one another’s emerging reflections, insights and ideas about the data. Any differences in the researchers’ interpretations were resolved through discussion, review of the supportive textual evidence for each theme, and consensus decision-making. Themes were ranked in strength based on a combination of the frequency with which they emerged and the intensity with which the speakers voiced the theme. 

### 2.6. Methodological Limitations

With regard to the on-site client-related data collection (i.e., interviews and questionnaires with clients), we are aware that the voluntary sampling approach could have resulted in biases in that clients with more positive views about their program would have been disproportionately inclined to take part in the evaluation study. Moreover, because there was a circumscribed number of days for each site visit and on-site data collection, we cannot be guaranteed that we achieved “saturation”, nor was the concept of saturation the means by which we determined the number of interviews to conduct at each site. At the same time, we have no reason to believe that clients who held less positive perspectives were disinclined to participate in the study, and the confidential, conversational approach to interviewing facilitated participants sharing their diverse experiences and perspectives. As well, given that there will be a second round of on-site data collection with clients, there will be an opportunity to explore the issue of sampling bias and determine whether saturation was achieved.

## 3. Results

### 3.1. Theory of Change

**Figure 2:**
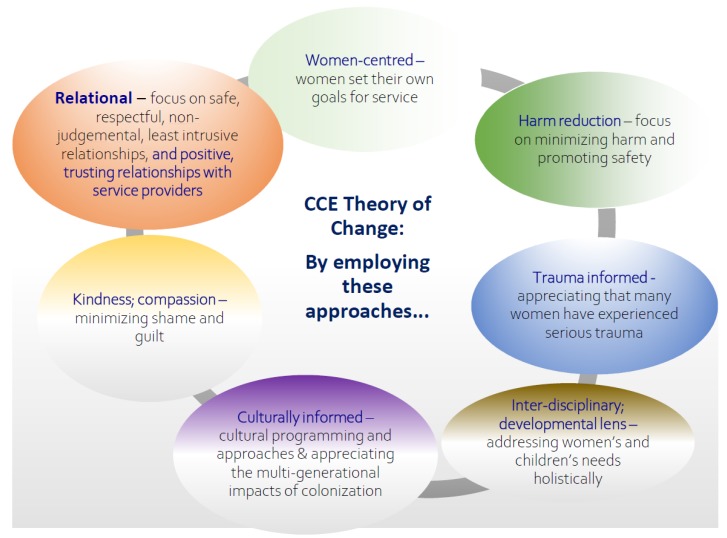
Key philosophy/approaches of programs involved in the Co-Creating Evidence: National Evaluation of Multi-Service Programs Reaching Women at Risk (CCE) project.

**Figure 3:**
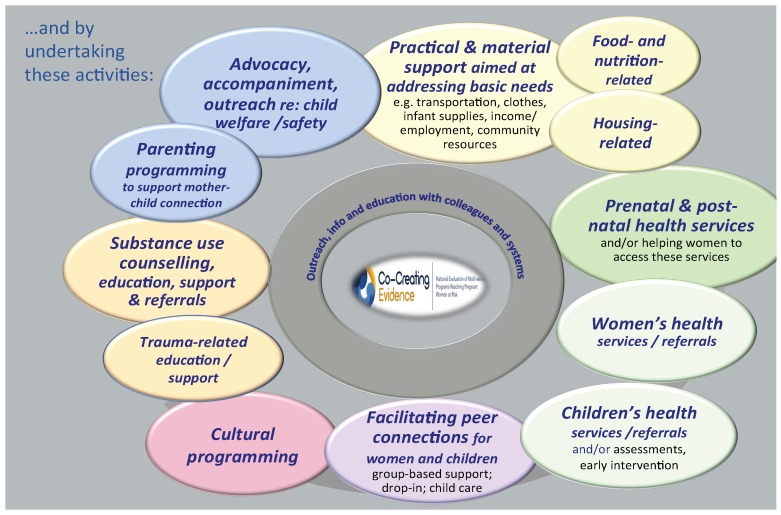
Key activities/services of programs involved in the Co-Creating Evidence project.

**Figure 4:**
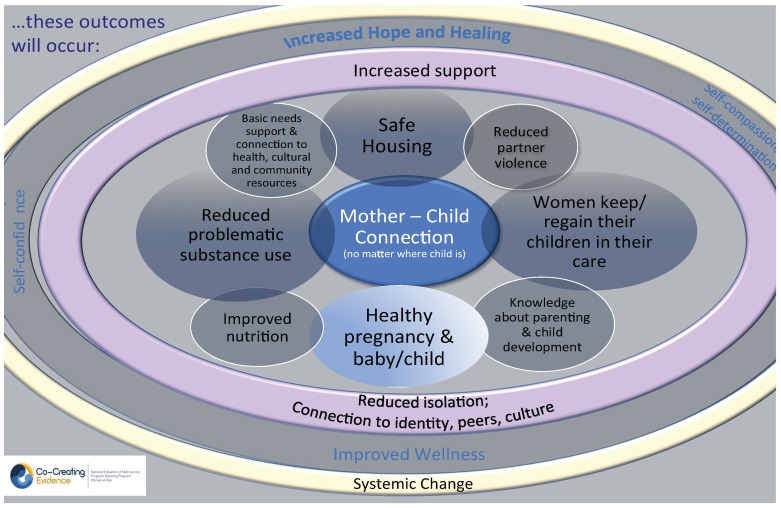
Anticipated outcomes of programs involved in the Co-Creating Evidence project.

In program evaluation, a Theory of Change is created to show the interconnections between the program’s philosophy/approaches; activities; and anticipated outcomes at the client/family, community, and systems levels. As part of the developmental phase of this project, the team and the Program Coordinators/Managers of the eight participating sites collaboratively developed a Theory of Change (depicted in Figure 2, Figure 3 and Figure 4); this was based on a framework for evaluation of FASD prevention and support programs [17]. In keeping with a collaborative and community approach to developing a Theory of Change, the CCE Theory of Change was created through multiple discussions during the initial face-to-face meeting with all programs in June 2017; following this, the research team revised and finalized the Theory of Change to incorporate all the Program Coordinators’ feedback. The CCE’s Theory of Change uses a social determinants of health lens that recognizes that pregnant women use substances for multiple reasons including for issues associated with trauma, violence, isolation, mental health, poverty, racism, and impacts of colonization [8,37].

### 3.2. Overview of Programs’ Governance, Services and Activities

Operationally, four of the eight programs in the study are under the aegis of a health authority (HA), and four are under the aegis of a community-based agency (CBA). As can be seen in Table 1, through a combination of their own staff or staff from partner organizations providing services on site, all programs offer a mix of health and social services and supports aimed at meeting clients’ health, social, cultural and practical needs. Nevertheless, programs connected to a health authority were more likely to offer access to health services on site (e.g., public health nurse, physician, nurse practitioner, or midwife). 

### 3.3. Client Characteristics

Based on the client data submitted by program sites, in the first two quarters of the study’s data collection period (April–September 2018), a total of 712 women participated in the eight programs. Table 2 shows the number of clients per program as well as other demographic characteristics by program site; as can be seen, the range between program sites for many client demographic variables was noteworthy. For example, over half of clients self-identified as being Indigenous (56% during Quarter 1 of data collection); however, this ranged from 97% at the Mothering Project in Winnipeg to 0% at Baby Basics in New Glasgow, Nova Scotia. With respect to clients’ age, the client database showed that the majority of women participating in these “one-stop” prevention programs were not young (i.e., under age 25); in fact, 37% were over 31. In addition, while most clients were pregnant at intake (84%, range 50–100%) and almost half were in their first trimester, not all women who participate in these programs are pregnant at intake; some were seeking postnatal assistance with other key issues such as housing, child welfare, and/or achieving goals related to reduced, safer, or quitting substance use. 

In addition, based on the client data at intake provided by program staff, 57% of clients were reported as actively using substances (21–97% across the eight program sites). Overall, 41% of clients across program sites were engaging in problematic substance use, defined as the use of substances that result in negative consequences in a person’s daily life, including adverse health consequences [38,39], and 19% reported being new to recovery (e.g., <3 months), with a range from 0% to 46% across sites. Further, 60% of clients were either homeless or had insecure or unstable housing (range across programs was 38–88%) at intake. Although the number of missing data items or “don’t know” responses was typically small (0–5%), there were a few notable exceptions; for example, Baby Basics (BB) had 71% “don’t know” responses for substance use. 

### 3.4. Clients’ Perspectives on Key Issues at Intake

In the qualitative interview, after discussing how they learned about their program and what they hoped to get from participating in it, clients were asked to describe their situation prior to becoming involved. The top themes that emerged included:

- Substance use issues—yet readiness for change
- Unsafe or inadequate housing
- Maternal–child separations and/or child welfare involvement
- Violence, trauma and/or intimate partner violence
- Limited social support and/or isolation
- Mental health issues

#### 3.4.1. Substance Use Intertwined with Other Issues, yet Pregnancy Sparks Readiness to Make Changes

Numerous women spoke of their problematic substance use and their desire to reduce or quit upon learning of their pregnancy; in addition, nearly all of these women also spoke of inadequate housing, violent relationships, mental health issues, maternal–child separations, and isolation (sometimes self-imposed in order to address their substance use issues). In these women’s words:

*I was pregnant. I quit using during the summer, but I was struggling with abuse, depression, anxiety and co-dependency.*

*I was pregnant but was determined to stay sober. My first daughter was apprehended at birth and was affected by alcohol, so I wanted to do it differently this time.*

*I was trying to quit doing drugs, hanging out with people using drugs. They were a bad influence. I was also pregnant with my sixth child; the five older children are all in care.*

#### 3.4.2. Housing Insecurity

As also indicated by Table 2, the majority of evaluation informants reported that before becoming a participant of their program, their housing was unsafe or inadequate; moreover, many women reported being homeless (or “hidden homeless”, e.g., couch-surfing) or were in the process of fleeing violent relationships. Women stated:

*I was working on the street and supporting my habit. I was homeless and couch-surfing.*

*I was living in a two-bedroom apartment with my mom, sister, two boys, my ex and sometimes my mom’s friends. There could be 13 people at a time in the apartment.*

*My partner didn’t respect my space—there was partying, and I was uncomfortable; the housing was unsafe. There was domestic violence.*

#### 3.4.3. Maternal–Child Separation

Another key theme in women’s stories was their experience of maternal–child separations, and specifically the removal of their infant or older child(ren) by child welfare authorities. For many of these women, becoming pregnant had fueled a desire for change related to their substance use and related circumstances and a hope of having their baby remain in their care. In these informants’ words:

*I was living in [supportive housing]; pregnant and then gave birth. I was depressed and wanted to address my drug and alcohol use. My son was apprehended at birth.*

*I had started using again. It wasn’t a good time; after I had the baby I dropped her off with (Child and Family Services)—I felt that I wasn’t able to care for my daughter and that it was for the baby’s own safety.*

*I was pregnant. I had just quit using drugs a month before that. I was living with my boyfriend’s parents at the time. I knew that I’d be better off here at [the program] if I wanted to get my kids back.*

#### 3.4.4. Violence, Trauma or Intimate Partner Violence

Many clients spoke of experiencing violence, trauma or intimate partner violence, which was inextricably linked to their mental health issues and substance use and also had given rise to child safety concerns and/or their child(ren) going into care. As well, several women reported having unsafe housing, were at high risk of homelessness, or were living in transitional housing because they were in the process of leaving their former partner.

*My partner assaulted me when I was two months pregnant. I have two older children and am a single parent with social anxiety, mental health issues and a long history of trauma.*

#### 3.4.5. Isolation/Limited Social Support

As noted previously, many women spoke of feeling isolated, disconnected and lacking support. For some, this was because for a long time they had not had a strong social support network, having perhaps grown up in foster care or without supportive family; for others, their sense of isolation was due to mental health and/or substance use issues; for others still, they moved to a new community where they knew no one in order to flee a violent relationship or unsafe living situation; or, lastly, some women realized they needed to isolate themselves or cut their ties with unhealthy people in their lives in the interest of achieving their goals related to reduced substance use and healing.

*I didn’t have a lot of social support. I have no family and was raised in foster care and by my grandmother who is now deceased.*

*I was living in an SRO, using crystal meth and heroin every day. I almost never left the SRO. I didn’t like to go out. I was very much shut off from other people.*

#### 3.4.6. Mental Health Issues

Lastly, while mental health issues were another key theme in women’s discussions of their situation prior to engaging with their program, in nearly all instances, the issues of substance use, violence, and mental health issues were fully intertwined.

*I was fleeing an abusive relationship. My mental health was poor. I was addicted and not taking care of myself.*

*I was starting to fall back into drug use. I was depressed, and I was scared of my behaviour.*

### 3.5. Clients’ Involvement in and Experience of Their Program’s Services/Activities

In the qualitative interviews, clients were asked to provide examples of how they engaged with various programs activities or services. Their comments, presented in Table 3, provide a more fulsome picture of the services listed in Table 1 and demonstrate how a “one-stop” approach to accessing health care, material and basic needs support, help with child protection issues, parenting groups and information, substance use counselling and groups, and cultural programming was utilized and valued by clients. 

### 3.6. Clients’ Experiences: What Clients Like Best about Their Program

As part of the qualitative interview, clients were asked what they liked best about their program. While many informants identified more than one theme in their response to this—and all other—open-ended questions, the top themes to emerge were:

- Staff and their approach—caring, non-judgmental, supportive and helpful
- Friendships, social and peer support, sense of community
- “One-stop”—multiple services in one place
- Homey, safe, healthy environment
- Help with child protection issues
- Positive impacts for my child—friendships and socialization skills

A brief discussion of these findings follows.

#### 3.6.1. Staff and Their Approach: Caring, Non-Judgmental, Supportive, and Helpful

The strongest theme of what clients liked best about their program, voiced by more than one-third of interview participants, was the staff and staff’s caring, respectful, and supportive approach. Time and again, women emphasized that staff’s non-judgmental and non-stigmatizing approach was extremely important as it both promoted trust and honesty in their relationship and also led women to feel “like a person”. As a related point, several people stated that staff at their program “never gave up” on them, which they appreciated greatly as it made them feel valued and worthwhile. Further, numerous clients praised staff of their program for being very helpful and often going “above and beyond” typical service provision. In clients’ words:

*The people that work at [the program], they aren’t judgmental. I feel like a person at [the program], not a nobody.*

*The staff—they give you endless support. When you tell them something, like if there’s been a slip, there’s no judgement. They’re very genuine. The right people work here.*

*[I like] that they never gave up on me. I was stubborn and had mood swings when I was coming off drugs. I really appreciate that they never gave up on me.*

#### 3.6.2. Friendships, Social and Peer Support, Sense of Community

The second strongest theme was that women most liked the friendships they had developed with other program participants and the support they gained through their relationships with women and staff. Women appreciated making connections with others who could relate to their experiences. Along these lines, a number of clients stated that they liked the sense of community or family that they experienced at the program, and one woman expressed that she liked “being able to mentor other women”, which was facilitated by the program’s drop-in and group activities.

*Definitely, the friends I’ve met here. […]The moms are both in recovery and are new moms. It’s easy to relate to them and to get along; we have things in common and have the same aspirations and common goals.*

*It feels like a family. The staff genuinely care about everyone. They always have time for you. I’ve made good friends at [the program].*

*The sense of community. That’s the biggest thing. When we come here—because we see and hear other people—we get a more in-depth relationship with people here.*

#### 3.6.3. “One-Stop”—Multiple Services in One Place

Another strong theme of what clients liked best was the program offering multiple inter-connected services and resources all “in one spot”; as one client stated: “everything I need is in one place”. This meant that women did not have to travel to get to appointments, which could be a formidable barrier to accessing services. Moreover, as clients pointed out, being able to obtain health and wellness services and basic needs support through a “one-stop” program made it easier to satisfy social workers’ expectations related to child welfare and custody. Clients stated:

*I don’t have to go far to get to a doctor. There are all kinds of different resources here. There is a welfare worker, a housing worker, the tax lady, as well as food to eat. It’s awesome. There’s also a Walking Group and a Boxing Group.*

*There are lot of services in one place, such as the public health nurse, the nurse practitioner, and a social worker, which makes it easier to meet the Ministry’s goals and expectations. I don’t have to travel far to get to my appointments. It’s very convenient and close to housing.*

In voicing appreciation for their program’s different services and resources, clients highlighted the program’s health and medical services, easy access to a social worker, as well as the practical and material support, including food, grocery vouchers, clothing, and infant supplies.

*I like that they are hands on with support. They have medical, counselling, and practical support; everything I need is in one place. And I like that it is a safe place and there is food.*

*I like that you can come here and get any support that you need—for example, I can get help getting welfare or with my addictions.*

#### 3.6.4. Homey, Safe, Healthy Environment

In keeping with the themes of caring and non-judgmental staff, and friendships and sense of community, another top theme of what women liked best about their program was that the environment felt “homey”, comfortable, and safe. Indeed, one woman spoke of feeling as though she could “relax” at the program—which was a contrast to how she felt in other situations or programs—while another deemed the program a “sanctuary” within a community that otherwise, to her, was mainly “dysfunctional”. Importantly, one woman also noted that the program was a place where she could find safety away from an abusive ex-partner.

*It’s a safe place for me no matter what is going on in my life—I won’t be judged by anyone. I can be myself. It is a place to connect with other women. I can be less guarded at [the program].*

*It’s a place to eat and relax and get away from my abusive ex. He can’t bug me at [the program].*

*The ladies—my peers—and the staff. And that it’s in the North End. There’s so much in the North End that’s dysfunctional, and the program here is the one thing that’s functional. It’s like a sanctuary.*

#### 3.6.5. Help with Child Protection Issues

Another key theme of what clients liked most about their program was the support they had accessed in relation to child welfare issues, in particular, in getting advocacy and support during meetings with child protection workers and/or in having supervised visits with their child(ren) on-site at the program. Similarly, one client expressed great appreciation for staff’s support when her children were removed.

*I had a meeting with program social worker who encouraged me to meet with CFS and even inspected my house to give me suggestions for what CFS would be looking for. So, when we met with the CFS worker, I was surprised at how well the meeting went.*

*Plus, they are willing to support parents with kids in care. My two daughters have been removed again. [The program] is a support for me, helping me to get my children back.*

*That they are always available to help, no matter how I am feeling and no matter what is happening in my life. For example, they helped calm me down when the kids were apprehended. They gave me confidence and kept me on the right track. They gave me a lot of support. I had my supervised visits with my kids two to three times a week at [the program].*

#### 3.6.6. Positive Impacts for My Child—Friendships and Socialization Skills

A final key theme of what women like best about their program was that their child(ren) benefited and gained skills and friendships, just as they did themselves. In women’s words:

*Plus, my daughter gets so much out of it.*

*The best thing about [the program] is the drop-in. You meet other women and learn from them. You make friends. Plus, children get to make friends.*

*I like that it is geared towards children too. At first, I didn’t want my son involved because I didn’t want him around drug users but then I saw that the focus included children and that they were seen and assessed through [the program]—that [the program] was beneficial for children as well.*

## 4. Discussion

The preliminary findings of the Co-Creating Evidence study confirm that the programs are reaching vulnerable pregnant and parenting women who are facing a host of complex circumstances including substance use. They are doing so in part by addressing the social environmental factors affecting women’s access to services (e.g., housing, childcare, and transportation issues) and the reasons for and correlates of their substance use, while providing low barrier access, all of which have been shown to be effective strategies in service delivery [4,5,8,40]. 

The interim findings provide additional confirmation and support previous research demonstrating that substance use, mental health, trauma, violence, child welfare involvement, and lack of adequate housing are most often co-occurring issues [1,2,4,5,41,42]. Frequently, though not exclusively, it was the intersection of these factors along with being pregnant and wanting to parent their infant that were motivating factors in women’s decisions to seek help. This again affirms that pregnancy can be a critical juncture in women’s lives and a time in which a multi-disciplinary, multi-dimensional, integrated approach can make a difference [2,5].

Along these lines, other studies have concluded that programs that adopt a trauma-informed and harm reduction perspective are more effective in reducing pervasive stigma and other barriers and engaging women in services [4,5,8,10,26]. The programs in the Co-Creating Evidence study were firmly rooted in these approaches as well as others, including being relational, culturally informed, attentive to the mother–child connection, and accepting of the pace and type of change women were able to make. In so doing, they became invaluable sources of support for their clients. Indeed, the non-judgmental approach to working with clients displayed by staff was a top theme voiced by women in terms of what they liked best about their program. That clients trusted staff was a signature achievement in itself given the barriers to service, as documented in the literature, that are typically encountered by women in similar circumstances and a general wariness often expressed toward traditional health care providers [40,43,44,45]. Women’s belief and trust in staff and their program was evident as well during the qualitative data collection interviews; this, coupled with a desire to “give back” to the community of women facing similar barriers to their own, were strong factors in clients’ decisions to take part in the study and to talk openly about sensitive or difficult aspects of their lives.

With respect to client characteristics, there was a wide range amongst programs in relation to the percentage of clients experiencing problematic substance use at intake. At some programs, problematic substance use was a primary eligibility criterion, whereas at others, an eligibility criterion was for pregnant or parenting women to be impacted by substance use—a subtle difference. One program did not use problematic substance use as a criterion at all. The three programs with the highest percentage of clients with problematic substance use were also geographically located in areas with high levels of poverty, drug and alcohol use, homelessness, violence, and mental illness. 

At nearly every program site, the percentage of women with unsafe or inadequate housing at intake was approximately the same as the percentage of women with problematic substance use or who were new to recovery at intake. Perhaps not surprisingly, maternal–child separations and involvement of child welfare/protection authorities represented a strong theme in clients’ lives, lending more weight to the assertion that an integrated, multi-service model is most effective when it comes to addressing the range of unique and varied needs of pregnant and parenting women with problematic substance use [7,8,10,14]. Consistent with the literature [40], the fact that programs offered multiple interconnected services and resources in one place and/or removed barriers including transportation was another top theme in terms of what clients liked about their program. 

Despite knowing more about what constitutes effective programming when working with pregnant and parenting women with problematic substance use, little has been written about the actual components of programs or how they are effective. A summary review of four community FASD prevention programs, prepared by Canada FASD Research Network’s Action Team on FASD Prevention from a Women’s Health Determinants Perspective noted that without practical assistance by way of nutritional and transportation supports, women cannot meet other goals such as reducing or stopping their substance use [46]. The Co-creating Evidence study supports these findings and contributes to knowledge about the components that are making a difference. In terms of practical supports, the availability of food/nutrition programming was frequently cited by clients as something that they appreciated. Whether it was a hot meal that was cooked on site or that clients helped to make as part of a drop-in group, or whether it was receiving nutritional supplements or groceries such as milk, eggs, and cheese, clients consistently voiced their gratefulness for this type of support. All types of food support were perceived as having financial as well as nutritional benefits, and meals that were part of programming brought social, emotional and skill development benefits for both women and their children. As with other forms of material support, programs’ help with basic needs, by way of donations of baby clothing and equipment, furniture, or gift cards that allowed clients to buy essentials for daily living, also provided financial relief for clients. 

Finding affordable, safe housing was a constant quest; the advocacy and proactive support provided by program staff, including connecting them with housing programs, helping to complete housing applications, meet with landlords, practice being interviewed for housing, and apply for income assistance and shelter allowance, helped many clients begin to find stability in their lives. Without housing, they were far less likely to satisfy child welfare authorities’ requirements for a safe environment or to address related custody or safety concerns. The handful of programs that had housing on-site (or in the case of one site, as a housing-based program), meant that clients had peace of mind in relation to two significant domains: accommodation along with proximity to a one-stop multi-service program that addressed all of their other social, medical, and health concerns. 

In terms of health services, in nearly all of the eight programs taking part in the current study, primary and/or prenatal care was an integral component of the program, delivered in-house or through partnership in the community. As such, clients availed themselves of a range of sources of pre-natal/post-natal health support (e.g., appointments with a midwife, physician, nurse or nurse practitioner; regular ultrasounds; transportation to appointments), health services for their own issues apart from pregnancy (e.g., birth control; regular check-ups; appointments for Suboxone; mental health medications), and health assessments for their children (e.g., development delays, speech, medical issues) as well as referrals for their children (e.g., dentists, pediatrician, Infant Development workers). Not surprisingly, the provision of a range of health services enabled clients to stay on top of their own health and wellness needs. For many this was a new experience, having been wary in the past of seeking health care services due to their association of prenatal care with being reported to child welfare authorities. Further, with a more positive and proactive relationship with health care practitioners, their children’s health and development issues were also potentially identified much sooner. Early identification is often key to mitigation of potential long-term negative impacts of development delays, dental deterioration, mental health, and other physical maladies. This aspect of programming—the provision of or linkages to primary health services—appears to distinguish the programs in the Co-Creating Evidence study from integrated treatment services for pregnant and parenting women, as a recent review of integrated services for similar clients in the province of Ontario found that primary and pre-natal care services were rarely named as partners, identifying a potential area for quality improvement of integrated treatment services [47].

The other primary activities that clients substantially valued were child welfare support and advocacy, substance use groups/programming, and peer/social connections. With respect to child welfare or custody, all but one program actively worked with clients to address child safety concerns, either by having their own social worker on-site who helped walk clients through meetings with provincial child welfare authorities, or through an arrangement with provincial child and family services to provide a social worker, who helped clients navigate extant child safety expectations and concerns.

Many clients expressed deep relief for the guidance they received, finding that it made it easier for them to ultimately address the safety concerns that were getting in the way of them parenting their infant or older children. Often clients were able, with the program’s support, to prove and improve their capacity as parents, thereby allowing child welfare services to close their files. With the active involvement of program staff, even clients who were unable to parent day-to-day were often able to establish or deepen a relationship with their child; programs often allowed supervised visits to occur on-site as a way of supporting the mother–child relationship. 

Peer relationships were also very important, as clients sought to build a different life and to develop new, healthier social connections for themselves and their children; for many women, the friendships and positive relationships formed through their program participation continued beyond the program’s hours and walls—e.g., through weekly walking groups and children’s birthday parties. Further, programs’ safe, non-judgmental environment resulted in women experiencing a positive sense of community and belonging. 

In terms of what makes the programs successful, from clients’ perspectives, there is no doubt that the combination of the right kind of staff, the right kind of approach, and multiple services in one location were significant factors. This too fits with the literature, which suggests that integrated, wraparound services that address the social determinants of health, offer a spectrum of services that meet a woman where she is at, and demonstrate a commitment to her and her child while adhering to obligations to safety, are practices that make a difference [48]. 

## 5. Conclusions

These programs reaching vulnerable pregnant and parenting women who face problems with alcohol and other health and social challenges are a key component of a comprehensive FASD prevention response. Evaluation research that identifies the range of services required and found helpful by women and service providers is critical to advancing the work of FASD prevention, and to health and social care system planning overall.

## Figures and Tables

**Table 1:** Services/activities offered by programs via staff or service partners or via referrals.

Service/Activity	Number of Programs Offering Service/Activity on Site via Program Staff or Service Partners	Number of Programs Linking Clients to Service/Activity via Referral to Service Partners	Total Number of Programs Offering or Facilitating Access to Service
Basic needs support	8	0	8
Child assessment and early intervention	5	2	7
Child care on site	7	0	7
Child health	6	2	8
Child welfare support	7	0	7
Cultural programming	5	1	6
Drop in; peer connection	8	0	8
Food; nutrition	8	0	8
Health; medical services	6	2	8
Housing	4	4	8
Life skills	6	1	7
Mental health; trauma	8	0	8
Outreach	6	0	6
Parenting programs	7	1	8
Prenatal; postnatal care	7	1	8
Substance use counselling	7	1	8

**Table 2:** Client characteristics by program site, at intake.

	HWH	SW	MW	HER	RH	MP	BTC	BB	Total
Number of clients	95	327	82	50	24	37	66	31	712
Age									
<16	1%	0%	0%	0%	0%	0%	0%	3%	0%
16–24	37%	16%	28%	26%	25%	49%	24%	94%	27%
25–30	39%	35%	48%	26%	54%	35%	30%	3%	35%
>31	23%	49%	24%	46%	21%	16%	44%	0%	37%
Pregnant	79%	92%	100%	96%	63%	65%	50%	68%	84%
Substance Use									
Problematic use	21%	52%	28%	62%	46%	70%	18%	3%	41%
Harm reduction	0%	21%	5%	10%	0%	27%	17%	7%	14%
In recovery/abstaining	41%	8%	35%	4%	4%	0%	36%	3%	17%
New to recovery	37%	17%	22%	0%	46%	0%	26%	0%	19%
Non-problematic use	0%	2%	5%	4%	0%	0%	0%	16%	2%
Housing									
Not housed/ homeless	33%	29%	12%	46%	50%	11%	12%	0%	26%
Unsafe/inadequate	32%	33%	38%	28%	38%	60%	26%	39%	34%
Safe and stable	36%	37%	43%	10%	13%	27%	42%	26%	34%
Indigenous (self-identified)	42%	67%	23%	81%	77%	97%	10%	0%	56%

*Note:* Categories do not always tally to 100% due to missing data or “don’t know” responses.

**Table 3:** Clients’ involvement in and experience of their program’s services.

Program Activity	Clients’ Experiences—in Their Words
Food/nutrition	*I do the Breakfast Club, and we always feel free to use the kitchen—there’s a client fridge. And they have feasts, and kids have food. They don’t serve junk food; it’s healthy.* *Breakfast is made by the moms. It helps to learn new recipes/ideas and to try new foods. There are multiple foods that I now include in my diet that I wouldn’t have prior to coming here.*
Housing/basic needs support	*They helped me get into social housing when I was pregnant; it is housing with rules. I had to stay sober for 18 months and then once I graduated I got to take over the lease myself.* *I get anxious when I have to call “official” people. They did a letter of support for me so I could access disability funding. They also helped with transportation (bus tokens), and they gave me a gift card so I could buy a bathing suit, so I could go swimming with my daughter.*
Pre/post-natal health support	*They got me connected to an OBGYN, nurses, ultrasound; they drove me to appointments and followed up with me to see if I went. If I missed the appointment, we rebooked.* *I have check-ups here and get prenatal vitamins regularly.*
Women’s health services	*I see the nurse practitioner or doctor for birth control. It is such a comfort to have someone I know and trust to put the IUD in for me. I have sexual trauma and could not go to a stranger.* *The public health nurse makes sure I am healthy, have a regular pap test, etc. She was key in making sure I had proper care when I broke my arm.*
Children’s health, assessment, referrals	*My sons had vaccinations with public health nurse and saw the dental hygienist. He is getting dental surgery soon as a result.* *We were referred to Sunny Hill hospital and introduced to a dentist for the children. The staff made sure the children were up to date for their immunizations and referred them to an Infant Development worker.*
Support/advocacy re: child welfare	*When I was pregnant, the program helped with our anxiety about meeting with the social worker, and then she closed our file. Without the program, they wouldn’t have closed our file as soon.* *From the beginning the program has been at all the meetings with me. Because of their presence, there were no hidden agendas in terms of what I had to do to get my children back.*
Alcohol and drug counselling, education, and support	*Addiction services come here; I had a referral for family treatment. There is lots of support for relapse.* *I just signed up for the SWAG (Struggling With Addictions Group) program. I feel grateful.*
Peer/social connections and groups	*That’s huge here. I think that’s the main focus for me right now. All the women who did prenatal together, now our children are all the same age.* *I have connected with two women who are sober and parenting. I like connecting with them.* *That is what happens with all of the groups and for the kids as well—they get peer/social connections too.*
